# Dual-Task Performance in GBA Parkinson's Disease

**DOI:** 10.1155/2017/8582740

**Published:** 2017-07-27

**Authors:** Karin Srulijes, Kathrin Brockmann, Senait Ogbamicael, Markus A. Hobert, Ann-Kathrin Hauser, Claudia Schulte, Jasmin Fritzen, Michael Schwenk, Thomas Gasser, Daniela Berg, Walter Maetzler

**Affiliations:** ^1^Department of Neurodegeneration, Hertie Institute for Clinical Brain Research, University of Tübingen, Tübingen, Germany; ^2^German Research Center for Neurodegenerative Diseases (DZNE), University of Tübingen, Tübingen, Germany; ^3^Department of Geriatrics and Clinic of Geriatric Rehabilitation, Robert-Bosch-Hospital, Stuttgart, Germany; ^4^Department of Neurology, Kiel University, Kiel, Germany; ^5^Network Aging Research, Heidelberg University, Heidelberg, Germany

## Abstract

**Introduction:**

Parkinson's disease patients carrying a heterozygous mutation in the gene* glucocerebrosidase* (GBA-PD) show faster motor and cognitive decline than idiopathic Parkinson's disease (iPD) patients, but the mechanisms behind this observation are not well understood. Successful dual tasking (DT) requires a smooth integration of motor and nonmotor operations. This study compared the DT performances between GBA-PD and iPD patients.

**Methods:**

Eleven GBA-PD patients (p.N370S, p.L444P) and eleven matched iPD patients were included. Clinical characterization included a motor score (Unified PD Rating Scale-III, UPDRS-III) and nonmotor scores (Montreal Cognitive Assessment, MoCA, and Beck's Depression Inventory). Quantitative gait analysis during the single-task (ST) and DT assessments was performed using a wearable sensor unit. These parameters corrected for UPDRS and MoCA were then compared between the groups.

**Results:**

Under the DT condition “walking while checking boxes,” GBA-PD patients showed slower gait and box-checking speeds than iPD patients. GBA-PD and iPD patients did not show significant differences regarding dual-task costs.

**Conclusion:**

This pilot study suggests that DT performance with a secondary motor task is worse in GBA-PD than in iPD patients. This finding may be associated with the known enhanced motor and cognitive deficits in GBA-PD compared to iPD and should motivate further studies.

## 1. Introduction

Heterozygous mutations in the* glucocerebrosidase (GBA)* gene represent the most common genetic risk factor for PD so far [1]. Moreover, it has been repeatedly shown that patients with such mutations (GBA-PD patients) present with a different phenotype than idiopathic Parkinson's disease (iPD) patients. For example, they suffer from an earlier age of onset and more rapid disease progression, including motor and nonmotor symptoms, such as cognitive, autonomic, and neuropsychiatric impairment [2–6].

However, it is not yet clear whether GBA-PD patients also differ from iPD patients regarding dual-task (DT) performance. Dual tasking—the performance of two tasks simultaneously—is accomplished multitudinously in one's daily routine. It is required, for example, when crossing a street while observing the surrounding traffic or when talking while walking. Malfunction of this performance can impair safe ambulation in complex natural environments and even have fatal consequences, such as falls. Almost half of the falls of PD patients are the result of trying to carry out two or more tasks simultaneously [7]. Associations between impaired DT and increased risk of a future fall in PD have been described recently [8]. The simultaneous performance of two motor tasks seems to be a valuable fall predictor, fitting well with patients' balance complaints when, for example, taking a cup out of the cupboard.

DT performance has also been used to analyze deficits of motor-cognitive interaction in PD [9]. As gait is not a fully automatic motor task but requires attentional performance and executive functioning [10], an analysis of gait under DT conditions can help detect motor-cognitive deficits. The present work aimed to evaluate whether the known differences in motor and nonmotor impairments between GBA-PD and iPD are also reflected in differences in DT performance.

## 2. Materials and Methods

### 2.1. Ethics

The study protocol was approved by the ethical committee of the Medical Faculty of the University of Tuebingen (number 49720091). All participants gave written informed consent.

### 2.2. Mutational Screening

Of the PD patients from across Germany who donated DNA to our biobank (https://www.hih-tuebingen.de/ueber-uns/core-facilities/biobank/) between 2006 and 2009 and agreed to genetic testing, the two most common mutations of the* GBA* gene (p.N370S, p.L444P) were screened. For detailed information, refer to Brockmann et al. [2].

### 2.3. Patients

Thirty-three GBA patients with one of the above-mentioned mutations were identified. All were contacted via mail and/or telephone. Eventually, eleven patients were included in this study. Twenty-two patients could not be investigated due to a degree of clinical impairment that prevented participation. To evaluate GBA-PD-specific features, eleven idiopathic PD patients (controlled to have none of the two* GBA* mutations) were matched for age, gender, and disease duration and were included in this analysis.

### 2.4. Clinical Assessment

PD was diagnosed according to the UK Brain Bank Society Criteria [11]. All assessments were performed in the dopaminergic ON state. Actual medication was assessed and Levodopa dose equivalency calculated [12] (see Table 1). The severity of motor symptoms was assessed using the motor part of the Unified PD Rating Scale (UPDRS-III) [13]. The Montreal Cognitive Assessment (MoCA) was used to screen for cognitive deficits, and a score of <26 out of 30 points was interpreted as indicating the presence of cognitive impairment [14]. By use of the Trail Making Test, cognitive flexibility and working memory were assessed [15]. Mood disturbance was assessed with Beck's Depression Inventory (BDI-II) [16].

### 2.5. Gait Analysis

All assessments were performed in a straight corridor at least 1.5 meters wide to allow free 20-meter walks. Gait analysis was performed using a wearable sensor unit (DynaPort Hybrid®, McRoberts, The Netherlands) attached via belt to the lower back. The sensor unit contained a triaxial accelerometer and a triaxial gyroscope. Data were transferred to McRoberts for automated gait analysis. Of the 20 m walked, the first and last 15% of the steps were excluded from the analysis to analyze only steady-state gait.

### 2.6. Single- and Dual-Task Procedure

All participants performed* three ST trials*: walking at a fast speed, checking boxes, and subtracting serial 7s. During the box-checking task, participants were instructed to mark as fast as possible each of the 32 boxes with a pencil on a paper sheet fixed on a clipboard held in their hand. During the subtracting task, subjects were asked to subtract serial 7s from a randomly chosen three-digit number until 10 subtractions were completed as fast as possible.

All participants then performed* two DT trials*: walking while checking boxes and walking while subtracting serial 7s. The following parameters were collected during the tasks: the duration of the tasks, the number of checked boxes, and the number of subtractions during DT. No instruction on prioritization (either walking or secondary task) was given.

### 2.7. Statistics

Statistical analysis was performed using JMP 11 software (SAS Institute Inc.). Clinical and demographic variables were compared nonparametrically using the Mann-Whitney *U* test (Table 1). Due to slight clinical differences between the GBA-PD and iPD groups (see Table 1), all outcome variables were corrected for UPDRS-III and MoCA by use of a multivariate regression model. Differences were considered significant at *p* < 0.05 (two-sided). Dual-task costs (DTC) were calculated using the formula according to [17, 18].

(1) $$\begin{array}{c} DTC=\frac{\left(ST-DT\right)}{ST}*100. \end{array}$$

DTC were defined as the percentage change between single- and dual-task performance: ([single-task − dual-task]/single-task) × 100. Therefore, DTC represent the relative difference in performance between ST and DT.

## 3. Results

**Figure 1:**
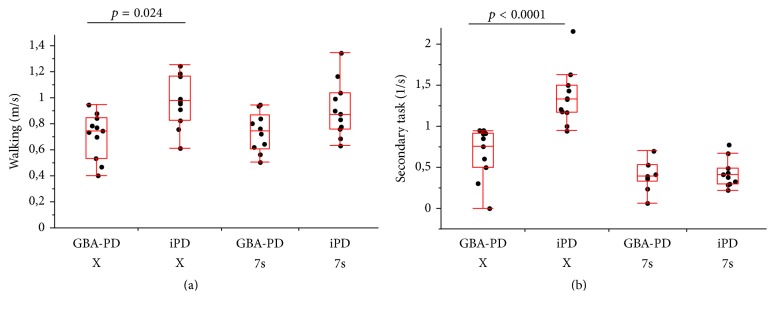
(a) Dual-task performance “walking while checking boxes (X) or while subtracting serial 7s (7s).” (b) Dual-task performance “checking boxes (X) or subtracting serial 7s (7s) while walking.” A logistic regression analysis, with the motor part of the Unified Parkinson's Disease Rating Scale and the Montreal Cognitive Assessment as covariables, including likelihood ratio was used to calculate *p* values. Significance level was set at *p* < 0.05. GBA-PD = Parkinson's disease patients carrying a heterozygous glucocerebrosidase mutation; iPD = idiopathic Parkinson's disease.

GBA-PD and iPD patients differed significantly regarding UPDRS-III scores (higher in GBA-PD; *p* = 0.01) and showed a trend towards a significant difference in the MoCA values (lower in GBA-PD, *p* = 0.06). Clinical characteristics are shown in Table 1. During the ST conditions, GBA-PD patients walked significantly slower than iPD patients (0.85 m/s versus 1.07 m/s, *p* = 0.04). The groups did not differ significantly regarding the speed of checking boxes and subtracting serial 7s. During the DT condition “walking while checking boxes,” GBA-PD patients showed a significantly slower box-checking speed (see below) and walking speed (0.75 m/s versus 0.97 m/s, *p* = 0.02) compared to iPD patients. Remarkably, the box-checking task under the DT condition showed a 100% separation of the groups (checking boxes while walking GBA-PD: 0.76 boxes/sec. (0.00–0.95); iPD: 1.33 boxes/sec. (0.95–2.17, *p* < 0.0001)). In the DT condition “walking and subtracting serial 7s,” walking speed and subtraction speed were not significantly different between the groups. Details are given in Table 2 and Figure 1. Although DT and ST differed between the groups, the DTC of all the speeds were not significantly different between GBA-PD and iPD. Detailed data are provided in Table 3. Sensor-based data of gait (steps, step time, cadence, double support time, and stride time variability) did not add relevantly to these findings (Supplementary Table in Supplementary Material available online at https://doi.org/10.1155/2017/8582740).

## 4. Discussion

GBA-PD patients are known to have more severe motor and nonmotor impairments compared to iPD patients [5]. This phenomenon was also observed in this small study. However, mechanistic aspects with respect to the specific deficits sheading some light on this phenomenon are not well investigated to date. This motivated us to investigate a specific and highly daily-relevant function on the interface of motor and cognitive performance, that is, multitasking. Results from this pilot study suggest that GBA-PD patients have indeed deficits with respect to this. This group had slower gait speed and box-checking speed under DT conditions (i.e., when performing two motor tasks), even after correction for “general” motor and cognitive deficits.

Previous studies of iPD patients [19–23] have shown that gait speed decreases when gait is simultaneously performed with a secondary task. Secondary tasks involving motor aspects may be more challenging than purely cognitive tasks in particular in patients with parkinsonism [8, 24, 25]. The results of the present study are in line with these findings: walking speed was reduced in both groups under the DT condition. Most notably, also the speed of checking boxes under DT was lower in GBA-PD than in iPD, and it differentiated the groups without any overlap (Figure 1). The findings and our conclusion should be interpreted with caution due to sample size but can motivate the investigation of the observed phenomenon in larger studies.

Importantly, DTC were similar in GBA-PD and iPD patients. The nature of DTC during gait is yet not fully understood. The results of fMRI studies suggest that cortical activity increases under DT conditions. Areas such as the cerebellum, the premotor area, the precuneus, and the prefrontal and parietal cortexes seem to be more active in iPD patients than in healthy individuals under DT [26]. The present results suggest that GBA mutational status does not have a relevant influence on DTC because it is possible that similar networks are activated under the DT condition in both PD groups. It seems that clinically more severely impaired GBA-PD patients show the capability to perform as well as iPD patients under DT conditions, though on a lower level. Whether this is due to pathophysiological differences or motor learning abilities has to be examined in future studies.

There is some evidence that there is a structural and even functional basis of our clinical finding. Cortical areas including the inferior frontal sulcus, middle frontal gyrus, and intraparietal sulcus have been reported to be involved in dual-task performance with increased activation of these areas under increased task complexity [27]. An MRI study [28] found more white matter changes (associated with more and more pronounced clinical deficits) in frontal and interhemispheric corticocortical connections of GBA-PD patients compared to nonmutation carriers and healthy control subjects. Other functional studies using PET showed hypometabolism in frontal and parietooccipital areas of GBA-PD patients [29, 30]. It is thus intriguing to hypothesize that the phenomenological deficit in dual-tasking performance presented by the GBA-PD patients is associated with the above-mentioned areas.

The effect observed while performing two motor dual tasks was not observed while performing a motor and a cognitive task (i.e., walking while subtracting serial 7s). This lack of difference may be best explained by an insufficient challenge of motor processing capacity. It has previously been hypothesized that (only) the use of the same neural capacities, for example, when performing two motor tasks, can exert group-specific differences [31]. Oscillatory dysfunction in basal ganglia due to dopamine depletion as well as reduced action selection due to dopamine deficiency could to some extent explain this phenomenon [32].

*Strengths and Limitations*. The small sample size is a limitation of this pilot study, and the reproduction of results in an independent and larger sample is required. Nevertheless, this study is, to the best of our knowledge, the first to present DT measures of GBA patients. All patients were carefully screened, recruited, and examined by specialists in the field of neurodegeneration. Furthermore, iPD patients, screened not to have a* GBA* mutation, were matched for age, gender, and disease duration to allow an adequate comparison between the groups. GBA-PD patients are known for having a more severe PD phenotype than nonmutation carriers do; therefore comparison of disease groups is challenging even when entirely new and daily-relevant parameters are assessed. Thus, we corrected all experimental results for UPDRS and MoCA scores. Further cognitive testing with, for example, the frontal assessment battery, could have added information about cognitive differences between the groups. We chose to examine dual-task performance instead, as it goes beyond the usual clinical investigation adding direct and daily-life-relevant information to our understanding of GBA-PD. We did not check for all pathologic GBA mutations due to logistic reasons. However, any pathologic mutation in our iPD cohort should weaken our results and thus does not argue against the correctness of our results. The DT procedures in this study have been successfully applied in previous work [33] and include different types of secondary (motor and cognitive) tasks.

## 5. Conclusions

GBA-PD show worse DT performance compared to iPD patients when executing two motor tasks simultaneously. If confirmed in larger studies, this pilot observation could be of relevance for clinical counselling.

## Supplementary Material

Supplementary Table: Qualitative gait parameters during single- and dual task gait, assessed using a wearable sensor unit.

## Figures and Tables

**Table 1:** Demographics and clinical characteristics.

	GBA-PD	iPD	*p* value
*Demographics*			
Male (female) [*n*]	9 (2)	9 (2)	1.00
Age [years]	58 (41–70)	62 (41–70)	0.51
Age of onset [years]	50 (28–65)	54 (36–62)	0.22
Disease duration [years]	6 (4–13)	6 (3–10)	0.46
Levodopa dose equivalent	700 (100–1500)	400 (80–800)	0.27
*Motor function*			
UPDRS-III (0–108)	35 (24–55)	27 (6–51)	**0.01**
*Nonmotor function*			
MoCA (0–30)	25 (11–29)	28 (23–30)	0.06
TMT A [s]	45 (30–263)	37 (25–66)	0.31
TMT B [s]	95 (47–300)	86 (50–300)	0.37
ΔTMT [s]	44 (16–98)	39 (24–234)	0.89
BDI-II (0–63)	9 (5–27)	9 (1–31)	0.49

Mann-Whitney *U* test. Values are given in median (range). Significance level was set at *p* < 0.05. UPDRS-III = Unified Parkinson's disease rating scale, part III motor score; MoCA = Montreal Cognitive Assessment; TMT = Trail Making Test; ΔTMT = TMT B − TMT A; BDI-II = revised version of the Becks Depression Inventory; GBA-PD = Parkinson's disease patients carrying a heterozygous glucocerebrosidase mutation; iPD = idiopathic Parkinson's disease.

**Table 2:** Single- and dual-task performance.

	GBA-PD (*n* = 11)	iPD (*n* = 11)	*p* value
*Single-task condition*			
Walking speed [m/s]	0.85 (0.54–1.13)	1.07 (0.71–1.35)	**0.038**
Checking boxes [1/s]	1.03 (0.53–1.84)	1.56 (0.91–2.29)	0.059
Subtracting [1/s]	0.31 (0.05–0.49)	0.31 (0.16–0.71)	0.134
*Dual-task condition*			
Walking speed while checking boxes [m/s]	0.75 (0.39–0.95)	0.97 (0.61–1.25)	**0.024**
Checking boxes while walking [1/s]	0.76 (0.00–0.95)	1.33 (0.95–2.17)	**<0.0001**
Walking speed while subtracting [m/s]	0.75 (0.51–0.95)	0.88 (0.63–1.35)	0.115
Subtracting while walking [1/s]	0.40 (0.07–0.71)	0.42 (0.23–0.78)	0.979

Values are given in median (range). A logistic regression analysis, with the motor part of the Unified Parkinson's Disease Rating Scale and the Montreal Cognitive Assessment as covariables, including likelihood ratio was used to calculate *p* values. Significance level was set at *p* < 0.05. GBA-PD = Parkinson's disease patients carrying a heterozygous glucocerebrosidase mutation; iPD = idiopathic Parkinson's disease.

**Table 3:** Dual-task costs.

	GBA-PD (*n* = 11)	iPD (*n* = 11)	*p* value
Walking while checking boxes [%]	11 (−11–48)	9 (−0.6–27)	0.88
Checking boxes when walking [%]	25 (−15–100)	12 (−10–44)	0.09
Walking while subtracting [%]	35 (27–59)	40 (33–52)	0.57
Subtracting when walking [%]	−39 (−121–13)	−16 (−78–39)	0.09

Values are given in median (range). A logistic regression analysis, with the motor part of the Unified Parkinson's Disease Rating Scale and the Montreal Cognitive Assessment as covariables, including likelihood ratio was used to calculate *p* values. Significance level was set at *p* < 0.05. GBA-PD = Parkinson's disease patients carrying a heterozygous glucocerebrosidase mutation; iPD = idiopathic Parkinson's disease.
