# Supplementary material for: Dual-Task Performance in GBA Parkinson's Disease
Source: Parkinsons Dis. 2017 Jul 27;2017:8582740. doi: 10.1155/2017/8582740 (PMC5551514; doi:10.1155/2017/8582740)
Supplement: Supplementary file 1 — Supplementary Table: Qualitative gait parameters during single- and dual task gait, assessed using a wearable sensor unit. [file 8582740.f1.docx]

Supplementary table: Qualitative gait parameters during single- and dual task gait, assessed using a wearable sensor unit

|  | **GBA-PD (n=11)** |  | **iPD (n=11)** |  | **p-value** |
| --- | --- | --- | --- | --- | --- |
| **Qualitative gait parameters during single task** | | | | | |
| Steps [N] | 21 (15-32) |  | 19 (15-27) |  | 0.47 |
| Step time [s] | 0.50 (0.31-0.54) |  | 0.45 (0.41-0.55) |  | 0.36 |
| Cadence [steps/min] | 120 (112-191) |  | 133 (110-145) |  | 0.66 |
| Double Support time [s] | 0.30 (0.21-0.38) |  | 0.28 (0.20-0.36) |  | 0.27 |
| Stride time variability, CV [%] | 10.1 (2.9-27.4) |  | 14.1 (2.3-21.8) |  | 0.93 |
| **Qualitative gait parameters during dual task (walking while checking boxes)** | | | | | |
| Steps [N] | 23 (17-35) |  | 19 (16-33) |  | 0.98 |
| Step time [s] | 0.53 (0.47-0.66) |  | 0.48 (0.31-0.61) |  | **0.04** |
| Cadence [steps/min] | 112 (92-127) |  | 126 (99-192) |  | **0.02** |
| Double Support time [s] | 0.35 (0.24-0.44) |  | 0.27 (0.18-0.38) |  | **0.04** |
| Stride time variability, CV [%] | 10.9 (2.6-15.5) |  | 8.2 (2.5-38.3) |  | 0.72 |
| **Qualitative gait parameters during dual task (walking while subtracting)** | | | | | |
| Steps [N] | 21 (18-31) |  | 19 (15-27) |  | 0.52 |
| Step time [s] | 0.56 (0.48-0.75) |  | 0.51 (0.43-0.62) |  | 0.06 |
| Cadence [steps/min] | 108 (80-125) |  | 118 (97-140) |  | **0.049** |
| Double Support time [s] | 0.36 (0.26-0.5) |  | 0.32 (0.24-0.38) |  | 0.06 |
| Stride time variability, CV [%] | 7.14 (1.51-16.16) |  | 3.02 (1.17-20.77) |  | 0.91 |
| **Dual task costs of qualitative gait parameters during dual task (walking while checking boxes)** | | | | | |
| Steps [%] | 9.5 (-32-63) |  | 4.3 (-6-60) |  | 0.55 |
| Step time [%] | 6 (-2-52) |  | 4.3 (-35-16) |  | 0.23 |
| Cadence [%] | 7 (-2-34) |  | 4 (-56-14) |  | 0.18 |
| Double Support time [%] | 25.7 (-11-52) |  | 5.5 (-30-36) |  | 0.16 |
| Stride time variability, CV [%] | 9.1 (-60-169) |  | 11.7 (-87-177) |  | 0.74 |
| **Dual task costs of qualitative gait parameters during dual task (walking while subtracting)** | | | | | |
| Steps [%] | 0 (-14-56) |  | 0 (-6-20) |  | 0.84 |
| Step time [%] | 8 (0-44) |  | 10.9 (-4-26) |  | 0.37 |
| Cadence [%] | 7.4 (-1-31) |  | 8.8 (-5.7-20.9) |  | 0.51 |
| Double Support time [%] | 15.4 (-21-86) |  | 10.3 (-19-68) |  | 0.79 |
| Stride time variability, CV [%] | 17 (-68-51) |  | 27 (-88-24) |  | 0.16 |

Values are given in median (range). A logistic regression analysis, with the motor part of the Unified Parkinson’s Disease Rating Scale and the Montreal Cognitive Assessment as covariables, including likelihood ratio was used to calculate p-values. Significance level was set at p < 0.05. GBA-PD = Parkinson’s disease patients carrying a heterozygous glucocerebrosidase mutation; iPD = idiopathic Parkinson’s disease.
